# Natural Graphite Spheroidization Phenomena in Arc Furnace Metallurgical Process for High-Silicon Cast Iron

**DOI:** 10.3390/ma18184397

**Published:** 2025-09-20

**Authors:** Marcin Stawarz

**Affiliations:** Department of Foundry Engineering, Silesian University of Technology, 7 Towarowa Street, 44-100 Gliwice, Poland; marcin.stawarz@polsl.pl

**Keywords:** nodular graphite, hypereutectic cast iron, crystallization process

## Abstract

Grey cast iron with spheroidal graphite has been known and widely used since the 20th century (since 1947). Numerous methods have been developed for the secondary metallurgy process to produce nodular graphite. Spontaneous crystallization of nodular graphite is known in foundry practice and other fields. Examples of cast iron with spheroidal graphite include pure alloys with low sulfur content and natural samples containing nodular graphite, formed by natural forces (meteorites and combustion ash). This article presents the results of two industrial experiments that led to the formation of nodular graphite precipitates without the addition of elements that promote spheroidization. Studies were carried out on high-silicon cast iron intended for corrosion-resistant castings. TDA, chemical composition analysis, light and scanning microscopy, EDS, X-ray spectroscopy, and digital image analysis were used to identify the nodular precipitates. The analyses confirmed the presence of nodular graphite precipitates, and known growth mechanisms were assigned to them. It is likely that deoxidation of the metal bath during the metallurgical process contributed to the spontaneous crystallization of graphite spheroids.

## 1. Introduction

In magnesium- or cerium-modified cast iron, graphite growth can occur via several mechanisms, which have been described in detail by Stefanescu et al. [1,2,3,4]. These are a function of local conditions at the solidification boundary, which are influenced by undercooling and supersaturation. The structure of the nodular graphite is assumed to be the result of several distinct stages [5,6,7,8]: stage I, nucleation and growth of graphite in liquid metal; stage II, growth during eutectic transformation by carbon through the austenite shell; stage III, growth during cooling to room temperature with decreasing carbon solubility in the austenite. In the initial stage, the problem of graphite nodule formation seems to be explained by the circumferential growth mechanism proposed by Sadoch and Gruzlesky [9] in 1975. These studies were confirmed by other researchers, and Stefanescu presented a summary of the results in his article [5]. This growth is believed to be caused by the movement of steps around the surface of the ball, growing in the (1010) direction. The crystal grows in different directions, which meet at a certain point, forming new, roof-like twin boundaries from which new steps arise. During growth, significant defects, called voids, can form. These can be found in both the central parts and outer regions of the graphite precipitates [5]. In the second stage of nodular graphite growth, radial growth of cylindrical columns or conical sectors occurs. This mechanism is based on the model of a cone or spiral originating from a common nucleus. The formation is based on the principle of positive wedge disclination. This mechanism was described by Stefanescu et al. [5]. They described the wedge formation process by subtracting a wedge from the basic hexagon, causing the graphene sheet to wrap around itself at a 60° angle. A similar mechanism, also described by Stefanescu [5], assumes that the (001) direction of each plate is twisted by 2° around the radius of the sphere, and the (001) directions of all platelets are orientated almost parallel to the radius, causing spiral growth of graphite from a common nucleus. Sometimes, the growth of cylindrical columns can be observed, which cannot be explained by the previous mechanisms. With sufficient magnesium content in cast iron, graphite platelets gather in clusters that produce polyhedral graphite blocks similar to those found in natural graphite [5]. In the third stage, during cooling, the solubility of carbon in austenite decreases, and carbon diffuses into the graphite particles. This causes further growth of the spheres [5]. Under unfavorable crystallization conditions, various forms of degenerate graphite can be formed: dendritic, spiky, exploded and chunky [9,10,11,12,13,14,15]. These have a negative impact on the mechanical properties of ductile iron, which deviates from the target spherical shape. In ductile iron subjected to cerium spheroidization, dendritic graphite may form during slow cooling (with a large casting wall thickness). Exploded degenerate graphite, as the name suggests, is the result of cracking caused by a high content of rare earth elements, excessive eutectic saturation, or a melting process of iron with too high purity. The probability of spiky graphite formation increases with a high content of despheroidizing elements such as bismuth, antimony, lead, and titanium [12].

The mechanism of graphite nucleation and growth has been described in numerous works [1,2,3,4,5,6,7,8], although this is not exhaustive, as there are many gaps in this area that will certainly be filled by scientific progress. It is known that spheroidal graphite precipitates occur naturally and spontaneously, without the participation of additives that promote spheroidization. A good example of observed spheroidal graphite precipitates is the result presented by Coey et al. They present the results of studies on the “Canyon Diablo” meteorite [16], which documented spheroidal graphite precipitates. Undoubtedly, these precipitates were formed as a result of specific conditions affecting the complex alloy, which was subjected to high temperatures, pressure, etc., after entering the Earth’s atmosphere. Other authors [9] proposed a mechanism for spheroidal graphite growth in a pure Fe-C-Si alloy, without the participation of spheroidizing elements. Adamczyk et al. [17] described the precipitation of spheroidal graphite in fly ash during the combustion process. The authors write that in a sample of unburned coal annealed at 3000 °C, various highly graphitised morphological forms were identified, such as spheroidal graphite, hexagonal plates, columnar plates, and films with serrated edges [17].

This article discusses an example of spontaneous spheroidization of graphite in a casting alloy intended as a starting material for the production of corrosion-resistant high-silicon cast iron [18]. The results described are based on multiple experiments (conducted under industrial conditions) and constitute an attempt to explain the causes of the occurrence of spheroidal graphite in the absence of elements promoting spheroidization and to describe its growth mechanism based on the latest theories of the graphite crystallization process.

## 2. Materials and Methods

The melting process were carried out under industrial conditions using an electric arc furnace (L-500 with a power of 350 kW and a capacity of 500 kg, SMS group GmbH, 41069 Mönchengladbach, Germany). The melts were part of a two-stage process for the production of silicon cast iron. The two-stage treatment of high-silicon cast iron is described in [19,20]. The test results presented in this paper refer to ingots marked 1 and 2, which were produced as a result of two separate preliminary melting processes carried out under industrial conditions. In both cases, low-sulfur steel scrap was used as charge material, the chemical composition of which is presented in Table 1. The metal bath was supplemented with ferrosilicon (FeSi75) to a content of approximately 15%, which was previously annealed as described in [20]. Synthetic graphite with a carbon content of 99.35% C was added to the metal charge. The liquid alloy was heated to a temperature of approximately 1350 °C. After slag removal, the prepared and melted material was drained into a casting ladle and then poured into a steel casting mold. Portions of liquid metal were taken from the casting ladle (using a casting spoon) to fill the TDA samplers. The chemical composition of the castings after the first production stage (initial melt) is presented in Table 2.

The cooling curves were recorded using a MultiCon CMC-141 device (Simex, limited liability company, Gdańsk, Poland) and Electro-Nite samplers with a NiCr-Ni type “K” thermocouple (QuiK-Cup system by Heraeus Electro-Nite Limited company, 2 Kombajnistów Street, 41-200 Sosnowiec Poland). The chemical composition of the charge scrap and ingots after the remelting process was analysed using a Leco GDS 500 spectrometer (model no. 607-500, Leco Corporation, 3000 Lakeview Ave, St. Joseph, MI, USA) and a CS125 carbon and sulfur analyser (Leco Corporation, 3000 Lakeview Ave, St. Joseph, MI, USA).

Metallographic examinations were performed using a Keyence VHX-X1 light microscope (Keyence International NV/SA Bedrijvenlaan 5, 2800 Mechelen, Belgium) and scanning electron microscopy. A Phenom Pro-X scanning microscope with an EDS system (Phenom-World B.V. Dillenburgstraat 9T Eindhoven, 5652 AM, The Netherlands) was used. The shape of graphite (according to ASTM A247-19 standard [21]) was described for 10 random measurement fields (for each sample) at least 50–100 graphite particles (for each sample area) using the shape factor given by Formula (1):

(1) $$\mathrm{Nodularity}\;=\;\frac{4\pi *A}{P^{2}}$$

where:

A—particle surface area,
P—particle circumference.

During image analysis, the empty spaces in the graphite particles were automatically filled.

To determine the phase composition, XRD studies were performed using a X’Pert PRO X-ray diffractometer by Malvern Panalytical, 7602 EA Almelo, the Netherlands) using filter radiation from a copper anode lamp and a PIXcel 3D detector on the diffraction beam axis. Measurements were performed using the step method. Measurement parameters were: angular range: 25–125 degrees; 2-theta step: 0.026 degrees; scan time per step: 100 s; and mean “K alpha” wavelength for the cobalt lamp: 1.7909 Å (0.179 nm).

## 3. Results

As a result of the preliminary melting process carried out under industrial conditions, two separate sets of results were obtained for ingots designated 1 and 2, respectively. The results of the analysis of these ingots are presented below.

### 3.1. TDA

**Figure 1:**
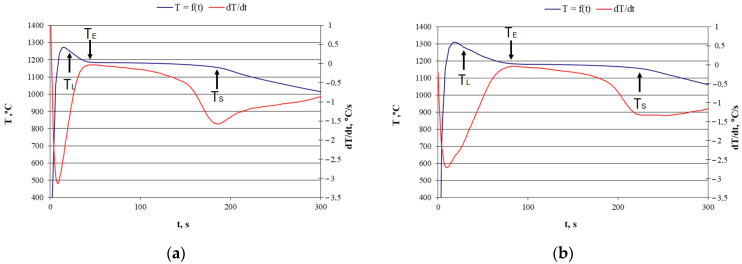
TDA curves: (**a**) casting No. 1; (**b**) casting No. 2.

The first stage of the research was to perform TDA for the two cases analysed. Figure 1 presents the recorded cooling curves along with the temperature-time derivatives. This enabled the determination of characteristic points in the crystallization process.

As a result of the TDA, characteristic process points were determined, such as the liquidus temperature T_L_, the solidus temperature T_S_, and the eutectic crystallization effect T_E_. A numerical comparison of the characteristic temperatures for both variants is presented in Table 3.

As can be seen, the T_L_ temperature values differ slightly by 6 °C. This difference is caused by the lower Si content in ingot 2 (14.57% Si) compared to ingot 1 (14.8% Si). As is known, silicon in cast iron lowers the liquidus temperature [22]. The difference in the TE eutectic crystallization temperature T_E_ is 2 °C, and the solid-state temperature T_S_ is the same. The main goal of this article is to describe the crystallization process of spheroidal graphite, which was identified in both ingots. In this respect, the analysis of both TDA diagrams did not give satisfactory results, because it is not possible to localize the thermal effects resulting from the crystallization of primary graphite. The thermal effects resulting from the crystallization of primary graphite should be visible in the case of hypereutectic cast iron [23,24]. Due to the low carbon content (approximately 0.6% C), the thermal effect resulting from primary graphite crystallization is not visible in the analyzed cases. It is possible that it will not be detected under industrial conditions.

### 3.2. Metallographic Analysis

**Figure 2:**
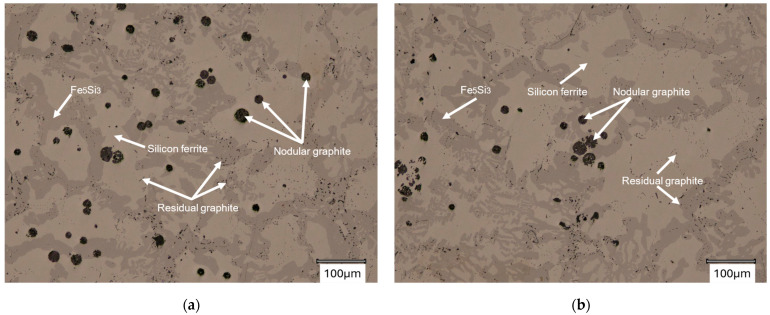
Casting microstructure with nodular graphite: (**a**) casting No. 1; (**b**) casting No. 2. Not etched.

**Figure 3:**
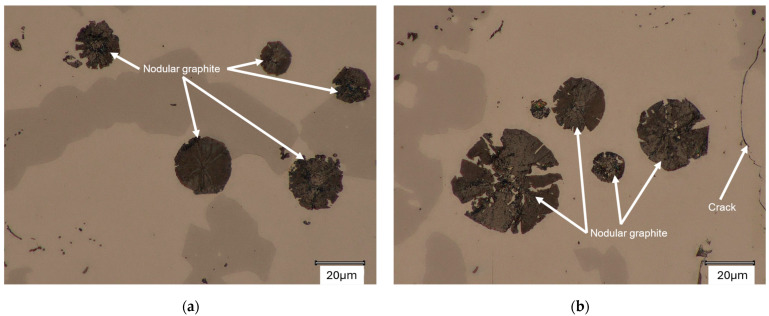
Casting microstructure with nodular graphite: (**a**) casting No. 1; (**b**) casting No. 2 Not etched.

In the first stage of metallographic analysis, samples from ingots 1 and 2 were observed under an optical microscope. Figure 2 and Figure 3 show the spheroidal graphite precipitates found in the ingots analyzed.

The spheroidal precipitates visible in Figure 2 and Figure 3 visually correspond to the graphite precipitates. Their detailed analysis will be conducted later in this paper. Residual graphite was also revealed in the microstructures (marked in Figure 2). The matrix microstructure of the analyzed alloys consists of silicon ferrite (light areas marked in Figure 3a,b) and a dark area (Fe5Si3 phase). Figure 3b also shows a crack in the sample. The material described in this paper is characterized by a tendency to develop gas porosity and cracks [18], hence the two-stage processing of the alloy intended for utility castings [20].

**Figure 4:**
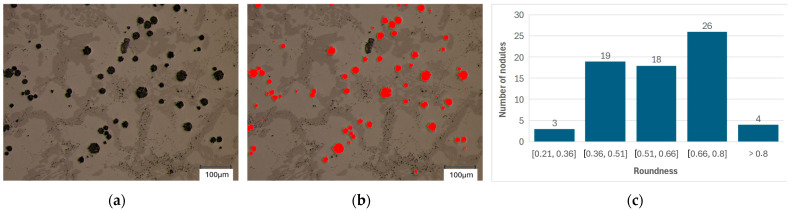
Analysis of the roundness of graphite precipitates: (**a**) microstructures of casting No. 1; (**b**) digital mask of graphite participations; (**c**) histogram.

**Figure 5:**
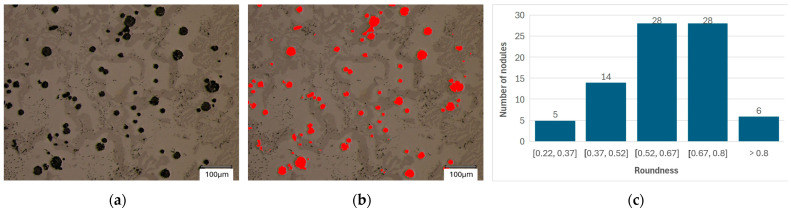
Analysis of the roundness of graphite precipitates: (**a**) microstructures of casting No. 2; (**b**) digital mask of graphite participations; (**c**) histogram.

In the next step, the shape of the spheroidal precipitates was analyzed. Figure 4 and Figure 5 present sample screenshots from the analysis along with the main graphs assigning the shape of the analyzed precipitates to individual classes.

The average results of the qualitative analysis of graphite precipitation are presented in Table 4. For sample 1, analysis was performed for 10 measurement fields, in which a total of 985 graphite precipitates were examined. For sample 2, an analysis was performed for 10 measurement fields, in which a total of 872 graphite precipitates were examined.

The partial results presented in Figure 4 and Figure 5 show that the sphericity distribution of the analyzed precipitates ranges from 0.2 to 1.0 However, a closer examination of the results reveals that most precipitates fall within the 0.5 class or higher. In this case, we can characterize the precipitates as spherical. The results of the statistical analysis (Table 4) indicate that the average sphericity values for both analyzed cases are 0.73 for sample 1 and 0.68 for case 2.

### 3.3. XRD Analysis

**Figure 6:**
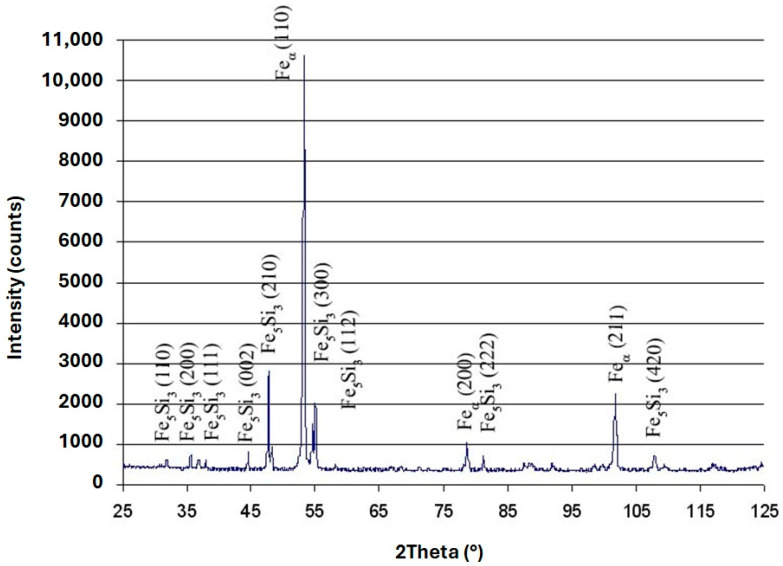
Diffraction analysis for casting No. 1.

Due to the significant similarity in the chemical composition of the alloys analysed, XRD analysis was performed only for ingot No. 1. The primary objective of this analysis was to determine the matrix type of the alloy tested. Similarly, the qualitative phase composition of the matrix of ingot No. 2 can be considered with 100% certainty to be homogeneous. X-ray diffraction analysis revealed the presence of silicon ferrite and an intermetallic phase of Fe_5_Si_3_ (98-063-3540) type. Detailed results are presented in Figure 6.

As can be seen in Figure 6, no effects are visible for carbon. This is likely due to distortion effects resulting from the presence of the Fe_5_Si_3_ intermetallic phase or the amorphous nature of the spheroidal precipitates.

### 3.4. SEM and EDS Analysis

**Figure 7:**
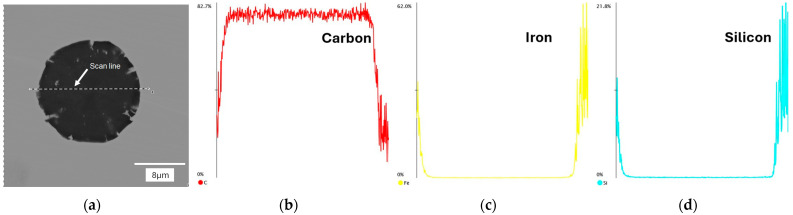
EDS analysis for nodular graphite: (**a**) microstructure; (**b**) carbon; (**c**) iron; (**d**) silicon. Casting No. 1.

**Figure 8:**
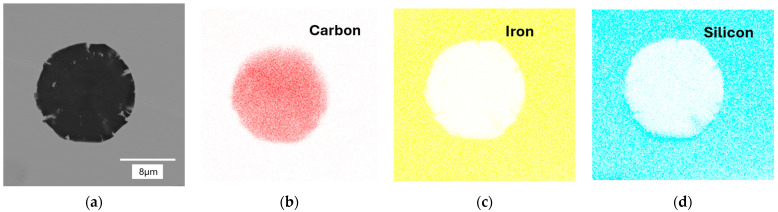
EDS map analysis for nodular graphite: (**a**) microstructure; (**b**) carbon; (**c**) iron; (**d**) silicon. Casting No. 1.

**Figure 9:**
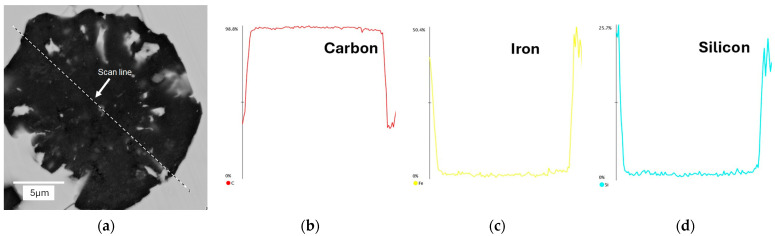
Linear EDS analysis for nodular graphite: (**a**) microstructure; (**b**) carbon; (**c**) iron; (**d**) silicon. Casting No. 2.

**Figure 10:**
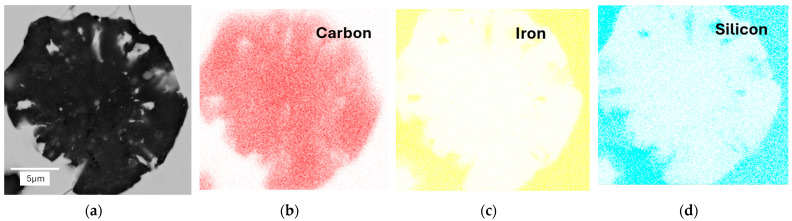
EDS map analysis for nodular graphite: (**a**) microstructure; (**b**) carbon; (**c**) iron; (**d**) silicon. Casting No. 2.

In the next stage of the study, scanning electron microscopy with EDS was used to identify spherical precipitates. Figure 7, Figure 8, Figure 9 and Figure 10 show the results of SEM analysis of selected spheroidal graphite precipitates, along with linear analysis and surface mapping using the EDS system.

Analyzing the results presented in Figure 7, Figure 8, Figure 9 and Figure 10, we can undoubtedly conclude that we are dealing with graphite precipitates. Of course, EDS analysis is subject to error due to the beam size (measurement energy), which affects the “depth” of the measurement. However, in the analyzed cases, elements highly enriched in carbon are clearly visible. The nature of these results clearly indicates graphite precipitates.

## 4. Discussion

The analyses carried out clearly indicate that the precipitation of spheroidal graphite in both melts discussed (carried out under industrial conditions) was unintentional. No elements promoting spheroidization (such as magnesium or RE) were introduced into the entire melting process. Furthermore, the entire high-silicon cast iron production process consists of two stages, and the paper describes the first stage of preliminary melting of the charge materials. The two-stage melting is intended to improve the quality of the final casting [20]. After the precipitation of spheroidal graphite in the starting material was detected, it was decided to repeat the experiment by melting another batch of steel scrap with the same chemical composition. Similarly to case 1, the precipitation of spheroidal graphite was obtained in ingot no. 2. In paper [9], the authors presented a concept of spheroidal graphite crystallization without the participation of spheroidizing elements. Their hypothesis applies to the pure Fe-Si-C alloy. Similar results were obtained in the experiment discussed, but for a completely different alloy, high-silicon cast iron. An additional difference is the purity of the resulting alloy. The results presented in this document refer to a process that takes place under industrial conditions in an electric arc furnace. The purity of the melt is not high as a result of the penetration of various types of impurities from the lining, metal residues from previous melts, etc. Therefore, the discussed chemical compositions contain elements that ultimately found their way into the chemical composition of the alloy. The ladle used to pour the metal into the metal ingot molds was also standard, used continuously throughout the production cycle. Therefore, the growth and crystallization conditions of spheroidal graphite in ingots 1 and 2 do not correspond to those described in [9].

**Figure 11:**
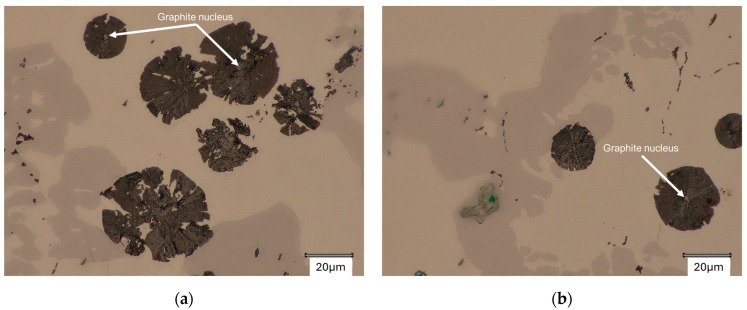
Graphite nucleus (stage I of the growth graphite process): (**a**) Casting No. 1.; (**b**) Casting No. 2.

Significant similarities were found with the spheroidal graphite growth pattern described in [1,2,3,4,5,6,7,8], primarily by Stefanescu. Microscopic observations of graphite precipitates in ingots 1 and 2 confirm these hypotheses. Figure 11 shows the visible stage I of the process, that is, the spheroidal graphite nuclei. Unfortunately, because of preparation problems and equipment limitations, it is currently impossible to identify the type of nuclei. Such studies are planned for the next stage of research.

**Figure 12:**
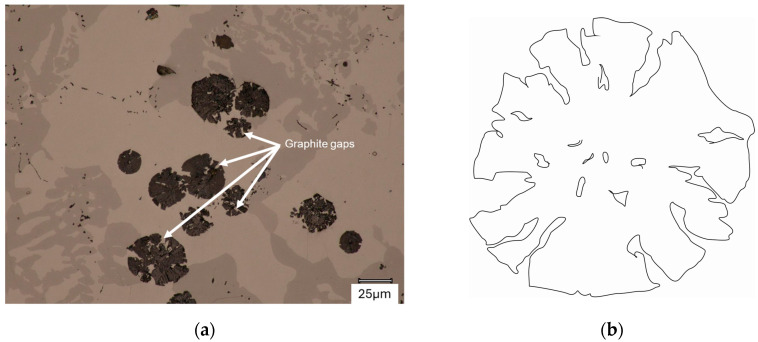
Graphite growth process (like as cabbage head): (**a**) Microstructure; (**b**) Schematic shape.

**Figure 13:**
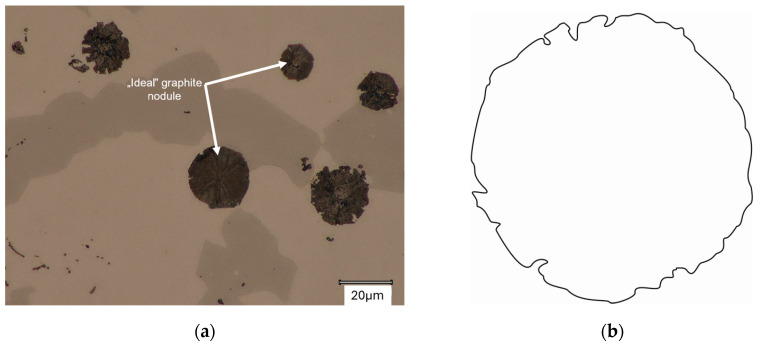
Graphite shape like ideal circle: (**a**) Microstructure; (**b**) Schematic shape.

The graphite growth pattern itself can also be described as circumferential (resembling a cabbage head), as described in [1,5]. This type of growth mechanism occurs in both cases analyzed. This is evidenced by the gaps in the structure of individual spheroids, described by Stefanescu [5] and shown in Figure 12. Figure 13 shows graphite particles with an almost perfectly circular shape.

**Figure 14:**
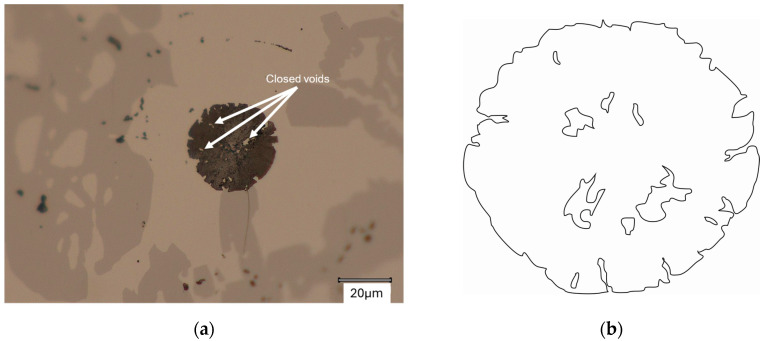
Graphite with closed voids: (**a**) Microstructure; (**b**) Schematic shape.

The graphite particles discussed here also contain closed voids scattered throughout the flat cross-section. An example of such a structure is shown in Figure 14.

The patterns of spheroidal precipitate growth presented in this paper are consistent with the data described in [1,2,3,4,5,6,7,8]. This also indirectly proves that we are dealing with spheroidal graphite precipitates as these data overlap.

A separate issue is the description of the graphite crystallization mechanism itself, without the involvement of elements promoting spheroidization. The sulfur content does not exceed 0.007%. This does not favour spontaneous spheroidization of graphite, as sulfur strongly inhibits it. It is suspected that other factors also influence the growth of graphite precipitates. Trace amounts of Ti could have deoxidized the metal bath, increasing the surface tension of the liquid, creating conditions for spheroidal graphite crystallization. The addition of Ti alloys to the metal bath in the production of vermicular cast iron is described in [25,26]. However, in the cases analyzed, we are dealing with trace amounts of Ti, which should under no circumstances be considered an alloying addition. Rather, it should be considered a contaminant produced during the industrial melting process, which has a deoxidizing effect. Another factor that creates special conditions is the overheating of the alloys in the arc furnace. High temperatures affect specific areas of the liquid metal [27,28,29,30], causing severe overheating and combustion of alloying components.

Observation of the structure of spheroidal graphite (studied in this work) reveals that its growth is not easy. This is indicated by various deformations and defects. In both cases, we are dealing with highly hypereutectic alloys. Experience in the metallurgy of cast iron indicates that primary graphite crystallizes directly from the liquid metal under such conditions. This was probably the case here. The difference is that the primary graphite from classical spheroidal graphite is much larger than that from other precipitates and generally smooth. In the cases discussed, it is true that the portion of graphite that has the characteristics of primary graphite can be separated, but only on the basis of its size. The morphological characteristics of the precipitates considered differ significantly from the ideal hypereutectic graphite precipitates [24].

## 5. Conclusions

Based on the test results of two high-silicon iron castings presented above, it can be concluded that:

- The described spherical precipitates are primary spheroidal graphite precipitates.
- The graphite spheroidization process occurred spontaneously, without the participation of elements promoting spheroidization.
- The graphite precipitate growth pattern corresponds to patterns [1,2,3,4,5,6,7,8] described by other authors. The circumferential growth pattern predominates.
- Some carbon in the alloy crystallized as fine precipitates of residual eutectic graphite.
- The likely cause of graphite crystallization was the melting conditions. The strong effect of the high temperature on the metal bath could have led to the removal of oxygen from the metal bath and an increase in the surface tension of the liquid, which promotes graphite spheroidization.
- The superheating effect refers to processes that occur near the melting electrodes, where the metal bath becomes very hot. Perhaps, this is a significant factor influencing graphite crystallization. Due to the complex dynamic phenomena occurring in this region, such as diffusion, oxidation, and so on, precise modelling of these phenomena will be difficult.
- The results of the industrial experiment described in this paper are reproducible.
- The alloys analyzed in this paper are cast iron. The carbon content does not exceed 0.6%, but the high Si content shifts the eutectic point of the alloy toward the lower carbon content. Both analyzed alloys crystallize with a eutectic transition.
- The thermal effect of primary graphite crystallization was not detected in the TDA plots because of the low carbon content of the alloy.

## Figures and Tables

**Table 1:** Initial chemical composition of steel scrap.

Chemical Composition, % of Weight														
C	Cr	Si	Mn	Ni	Mo	S	Cu	Al	P	Pb	Nb	Sn	Ti	Fe bal
0.080	0.007	0.001	0.594	0.024	0.02	0.006	0.07	0.006	0.016	0.002	0.031	0.009	0.001	99.13

**Table 2:** Casting chemical composition after first melting stage.

Chemical Composition, % of Weight											
No.	^1^ Si	^2^ C	^2^ S	P	Mn	Mo	Cu	Mg	Ti	Fe bal	^3^ CE
1	14.8	0.565	0.007	0.042	0.305	0.019	0.064	0.00	0.025	84.173	5.02
2	14.57	0.611	0.006	0.041	0.329	0.01	0.069	0.00	0.022	84.362	4.99

^1^ Si analysis by weight method, ^2^ Carbon and sulfur analysis by CS 125 Leco, ^3^ CE = C + 0.3Si + 0.36P.

**Table 3:** Characteristic temperatures for TDA analysis.

°C			
No.	T_L_	T_E_	T_S_
1	1264	1187	1158
2	1270	1185	1158

**Table 4:** Nodularity analysis of the precipitates - statistical analysis.

Sample		Nodularity	Area, µm^2^	Circuit, µm	Max Diameter, µm	Min Diameter, µm	Feret Diameter (Horizontal), µm	Feret Diameter (Vertical), µm
1	Avg.	0.73	456.27	88.06	28.11	21	26.41	24.61
	Std.	0.16	342.57	56.81	18.53	6.66	18.39	8.2
	Max.	0.94	2600.12	511.96	209.65	40.03	210.4	53.56
	Min.	0.12	121.17	41.98	12.83	9.18	9.95	12.24
2	Avg.	0.68	415.93	87.56	27.55	19.7	25.63	23.39
	Std.	0.18	368.13	56.85	17.72	7.31	16.9	9.54
	Max.	0.93	2603.63	486.21	208.98	49.37	208.87	84.16
	Min.	0.11	118.83	42.88	13.54	5.32	9.95	9.18

## Data Availability

The original contributions presented in this study are included in the article. Further inquiries can be directed to the corresponding author.
